# A Cold Environment Aggravates Cough Hyperreactivity in Guinea Pigs With Cough by Activating the TRPA1 Signaling Pathway in Skin

**DOI:** 10.3389/fphys.2020.00833

**Published:** 2020-08-27

**Authors:** Ran Dong, Tongyangzi Zhang, Weili Wei, Mengru Zhang, Qiang Chen, Xianghuai Xu, Li Yu, Zhongmin Qiu

**Affiliations:** Department of Pulmonary and Critical Care Medicine, Tongji Hospital, Tongji University School of Medicine, Shanghai, China

**Keywords:** neurogenic inflammation, chronic cough, cold air inhalation, cold environment, cough reactivity, transient receptor potential ankyrin 1, guinea pigs

## Abstract

Cough exacerbation in cold environments is a characteristic feature of patients with chronic cough. There is consensus that inhalation of cold air stimulates cough receptors but this idea is not consistent with the fact that cold air is usually unable to directly enter the lower airway. To elucidate the effects of cold environments and transient receptor potential ankyrin 1 (TRPA1) on cough, we compared cough reactivity, airway inflammation, and TRPA1 expression in guinea pigs with chronic cough induced by the repeated inhalation of citric acid for 15 days. The guinea pigs were exposed to cold environments for three consecutive days from day 13 to 15. Repeated inhalation of citric acid increased cough reactivity to inhaled cinnamaldehyde. We found that exposure to cold environments further aggravated cough hyperreactivity in guinea pigs with chronic cough, but not in normal guinea pigs. Cough hyperreactivity was promoted when the whole body and trunk-limbs, but not the heads, of the guinea pigs were exposed to cold environments, and abolished by pretreating the skin through immersion in the TRPA1 antagonist, HC-030031. Substance P levels in bronchoalveolar lavage fluid, and TRPA1 expression in the trachea and skin, were increased in guinea pigs when the whole body and trunk-limbs, rather than the head, were exposed to cold environments. However, this trend was also abolished by pretreatment of the skin via immersion in HC-030031. Similar changes in TRPA1 expression were also detected in the sensory fibers of the trachea and skin, as identified by immunofluorescence and laser-scanning confocal microscopy analysis. These results suggest that exaggerated cough hyperreactivity induced by cold environments may be related to activation of the cold-sensing TRPA1 signaling pathway in the skin, rather than the inhalation of cold air.

## Introduction

Cough is a common symptom that often leads patients to seek medical care (Irwin et al., 1990; Lai et al., 2013). The management of cough remains a challenge (Gibson et al., 2016), and a chronic cough lasting for more than eight weeks can be an unpleasant experience for patients, with a profound and negative impact on their daily work and health-related quality of life (French et al., 2004; Ma et al., 2009). Despite the effects of this common symptom, the mechanisms underlying a cough have still not been fully elucidated.

Cough in patients with chronic bronchitis or chronic obstructive pulmonary disease is easily exacerbated in winter by cold weather but spontaneously relieved in summer when the climate is warm (Huang et al., 2015). Chronic cough caused by various etiologies is ubiquitously characterized by aggravation in a cold environment (Cho et al., 2003; Kanemitsu et al., 2016). It is generally accepted that inhalation of cold air decreases the temperature in the lower airways, activating cough receptors that are sensitive to thermal stimuli (Banner et al., 1985; Cho et al., 2003). However, cold air cannot enter the lower airways directly under normal conditions because of the optimal heating and humidification of inhaled airflow through the nose. Currently, no evidence of impairment of the heating and humidification of the upper respiratory tract in patients with chronic cough exists. Therefore, the inhalation of cold air cannot be responsible for cough exacerbation in a cold environment in patients with chronic cough, unless the protective mechanisms of the upper respiratory tract are bypassed by open-mouth breathing (McFadden and Pichurko, 1985).

Humans and mammals perceive changes in ambient temperature and regulate their responses through thermoreceptors on afferent neurons that innervate the skin and mucosa. These receptors belong to a family of transient receptor potential (TRP) cationic channel proteins, the activation of which induces calcium influx and subsequent action potentials and impulses (Zheng, 2013). Transient receptor potential proteins are also widely involved in physiological and pathological processes, including pain transduction and neurogenic inflammation. Among the TRP channel subtypes, TRPV1, TRPV2, TRPV3, and TRPV4 respond to heat signals, while TRPA1 and TRPM8 are sensitive to cold stimuli (Patapoutian et al., 2003; Ferrer-Montiel et al., 2012). Furthermore, TRPV1, TRPA1, and TRPM8 are also expressed on vagal C and A-delta afferent fibers in the airways and are associated with the cough reflex (Grace et al., 2014). The TRPV1 protein plays an important role in capsaicin-induced and citric acid-induced cough but has no cold sensory function and is not directly related to cold-induced cough (Couto et al., 2013). Although TRPM8 is a cold sensor, most studies have shown that its agonists can inhibit the cough reflex (Millqvist et al., 2013). In contrast, inhalation of the TRPA1 selective agonist can induce cough in healthy volunteers and guinea pigs, while selective TRPA1 antagonists can inhibit cough (Birrell et al., 2009). Furthermore, TRPA1 is associated with the release of neuropeptides, such as substance P (SP), from airway C-fiber afferents, and airway neurogenic inflammation (Geppetti et al., 2010). Both SP and neurogenic inflammation in the airway underlie the pathogenesis of cough.

Based on the above information we hypothesized that in the context of cough hypersensitivity, cough exacerbation in cold environments is mediated by the activation of afferents sensing cold stimuli in the skin, rather than cold air inhalation, with TRPA1 as a key signal. In the present study, we investigated the underlying mechanisms of a cough and evaluated the effects of cold environments on cough reactivity to inhaled cinnamaldehyde (a TRPA1 agonist) in guinea pigs.

## Materials and Methods

### Main Reagents

All chemicals (except for antibodies) were purchased from Sigma-Aldrich (St. Louis, MO, United States). Citric acid, cinnamaldehyde, and pentobarbital were freshly prepared with physiological saline before use. The citric acid (4.2 g) was dissolved in 50 mL of physiological saline to prepare a 0.4 M solution. Cinnamaldehyde (3.776 mL) was mixed with 3.112 mL Tween 80, and then 3.112 mL ethanol, to make a 3 M stock solution, which was stored at −20°C and diluted with physiological saline to make the final working solutions of 20 mM daily. The TRPA1 antagonist HC-030031 (100 mg), was dissolved in 28.138 mL ethanol to make a 10 mM stock solution, which was diluted with ethanol to prepare 0.4, 0.8, and 1.6 mM solutions.

### Animals

Male Dunkin-Hartley guinea pigs (300–400 g) were provided by Shanghai Jiesijie Laboratory Animal Technology (Shanghai, China) and housed in conditions with controlled temperature (22 ± 2°C), humidity (50 ± 20%), and lighting (6:30 a.m.–6:30 p.m.). Food and water were freely available for at least 1 week before the experiment commenced in the Laboratory Animal Unit of Tongji Hospital, Tongji University School of Medicine. All study procedures were approved by the Ethical Committee for Care and Use of Laboratory Animals of Tongji Hospital and were in strict accordance with the principles and guidelines of the National Institutes of Health Guide for the Care and Use of Laboratory Animals.

### Citric Acid-Induced Cough Model in Conscious Guinea Pigs

The cough model was established by subjecting the guinea pigs to repeated inhalation of citric acid, as described previously (Xu et al., 2018). Awake animals were placed in a whole-body plethysmograph (EMKA, Bourré, France), where they were permitted to move freely, and then exposed to 0.4 M aerosolized citric acid for 3 min, twice daily to induce cough (Supplementary Figure S1A). The aerosol was generated via a jet nebulizer (ANP-1000; EMKA, Bourré, France) and delivered to the body plethysmograph at an output of 0.5 mL/min, with a median diameter of the particles between 0.5 and 2 μm. The cough module of the EMKA system, a respiratory flow analyzer (iox2.9 analysis system, EMKA, Bourré, France), was used to monitor the number of coughs during the inhalation of citric acid, by detecting the changes in the pressure waveform and sound waveform.

Control animals inhaled 0.9% sterile saline instead of citric acid. A previous study showed that repeated inhalation of 0.4 M citric acid for 25 days increased cough in a time-dependent manner and reached a peak on the 15th day, with a corresponding pattern in airway neurogenic inflammation, as indicated by similar changes in SP and calcitonin gene-related peptide in the airways over time (Xu et al., 2018). Taking this into account, we established a cough model for the guinea pigs that involved repeated inhalation of citric acid for 15 consecutive days.

### Exposure of Guinea Pigs to a Cold Environment

The guinea pigs were exposed to a cold environment from day 13 of this 15 day model. Exposure started 30 min after the inhalation of citric acid on the afternoon of the 13th day. This exposure lasted for 8 min daily, over three consecutive days (Supplementary Figure S1A). The cold environment was established in the variable temperature compartment of a refrigerator (Haier, China), set to 0°C, and monitored by a thermometer (DT-1, Jingchuang Electronics Co. Ltd, Jiangsu, China). Animals were placed in an airtight double-chambered body plethysmograph (EMKA, Bourré, France), with the head in the head chamber and the trunk-limbs in the body chamber. The guinea pigs were exposed to the cold environment by three different means, i.e., whole-body exposure, trunk-limb exposure, and head exposure. These modes of exposure were designed to imitate the respective conditions of, whole-body cold exposure with cold air inhalation, whole-body exposure without cold air inhalation, and cold air inhalation alone (Supplementary Figures S1B–D).

For whole-body exposure, guinea pigs were restrained in the double-chambered body plethysmograph, which was placed in the variable temperature compartment of the refrigerator, where the temperature was maintained at 0°C. For trunk-limb exposure, only the body chamber of the double-chambered body plethysmograph was placed in the variable temperature compartment of the refrigerator. However, the head chamber remained outside of the refrigerator and was exposed to room temperature (25°C). This was achieved by opening the refrigerator door and wrapping an airtight insulation layer of cystosepiment around the outer line of demarcation between the head and body compartments of the body plethysmograph. Head exposure was performed similarly to trunk-limb exposure, but with only the head chamber exposed to 0°C. The control animals were exposed to room temperature (25°C).

On exposure to the cold environment (0°C), the skin temperature of the exposed parts of the guinea pigs was stably decreased to 9.5 ± 0.1°C, with a corresponding temperature of 32.9 ± 0.1°C in the upper airways and 31.6 ± 0.3°C in the trachea (Supplementary Figures S2A–C). On exposure to room temperature (25°C), the skin of the exposed parts was maintained at 34.5 ± 0.4°C, as monitored by a thermometer. All experimental conditions were optimally selected based on the results of our preliminary experiments. No guinea pigs died or became debilitated during the study.

### Pretreatment With the TRPA1 Antagonist Before Exposure to the Cold Environment

To demonstrate the role of TRPA1 in the skin of guinea pigs exposed to a cold environment, we used the selective TRPA1 inhibitor, HC-030031, to treat the skin of the trunk and limbs immediately after inhalation of citric acid and 30 min before exposure to the cold environment (Supplementary Figure S1A). Conscious guinea pigs were restrained in the double-chambered body plethysmograph. The body chamber was immersed either in a vehicle solution or 1.6 mM HC-030031 solution for 5 s and 30 min later, exposed to 0°C in the same manner in which trunk-limb exposure was conducted. Our preliminary experiment showed that the effect of HC-030031 was dose-dependent, within the range of 0.4–1.6 mM with an optimal concentration at 1.6 mM, at which HC-030031 did not affect targets since cough response to inhaled capsaicin was not influenced (Supplementary Figure S3). The optimal immersion time was 5 s, within a range of 5–15 s tested in a preliminary study. The vehicle presented no detectable effects (Supplementary Figure S4).

### Cough Reactivity to Cinnamaldehyde in Awake Guinea Pigs

The preliminary study showed that inhaled capsaicin effected no change in the cough reactivity of guinea pigs following exposure to a cold environment (Supplementary Figure S3). These findings are consistent with those of Yoshihara et al. (1996). Therefore, we used cinnamaldehyde, a TRPA1 agonist, to replace capsaicin as a tussive agent. Cough reactivity to inhaled cinnamaldehyde was measured immediately after the guinea pig’s last exposure to the cold environment, according to previously described protocols (Liu et al., 2001), with minor modifications. Conscious guinea pigs were placed in the whole-body plethysmograph, where they were able to move freely, and were exposed to 20 mM aerosolized cinnamaldehyde for 5 min, delivered via a nebulizer (ANP-1000, EMKA, Bourré, France) with an output of 0.5 mL/min. The changes in respiratory pressure waves and breathing patterns were monitored by analog recordings and a respiratory flow analyzer (iox2.9 analysis system, EMKA, Bourré, France), as described above. Moreover, cough sounds were detected by a microphone installed on the roof of the body plethysmograph. Cough was automatically measured according to typical changes in cough pressure, and the sound waveform was measured by a respiratory flow analyzer (iox2.9 analysis system, EMKA, Bourré, France). The operators, who did not know the experimental procedures, observed the characteristics of cough in the animals, excluding sneezing and other irregular physical activities that the analyzer may have misdescribed. Cough reactivity was indicated by the number of coughs evoked during 5 min of cinnamaldehyde inhalation.

### Collection of Bronchoalveolar Lavage Fluid and Tissue Specimens

After the measurement of cough reflex reactivity to cinnamaldehyde, the guinea pigs were euthanized with an overdose of pentobarbital. The bronchoalveolar lavage fluid (BALF) was collected by injecting a 10 mL aliquot of sterile phosphate-buffered saline (PBS), precooled on ice, into the lungs three times, and withdrawn with gentle suction via an intratracheal polyethylene cannula. A mean volume of 8 mL was recovered from each animal. The recovered BALF was immediately centrifuged for 10 min at 1500 rpm (Thermo Fisher, Waltham, MA, United States). The supernatants were stored at −80°C for the enzyme-linked immunosorbent assay (ELISA), and the resultant precipitate was smeared and treated with the Wright-Giemsa stain. Differential cell counts were determined by counting at least 200 cells, according to standard morphologic criteria. Immediately after the collection of BALF, the intact trachea (length, 0.8–1.0 cm) and two pieces (1 cm^2^ each) of skin on the backs of the guinea pigs (shown in Supplementary Figure S5) were excised and either fixed in 4% paraformaldehyde at room temperature (25°C) for 24 h or immediately placed in liquid nitrogen, and then stored at −80°C until further processing. We selected the skin on the back because our preliminary study revealed that this area of skin showed the most abundant expression of the TRPA1 protein (Supplementary Figure S5).

### Immunohistochemical Analysis

Immunohistochemistry was conducted to analyze the expression and distribution of the TRPA1 protein in the skin and trachea. Fixed specimens of the skin and trachea were embedded in paraffin and sectioned into slices 5 μm in thickness, then dewaxed in xylene and rehydrated. Endogenous peroxidases were inhibited with 0.5% hydrogen peroxide in methanol for 10 min, followed by overnight incubation at 4°C with a rabbit polyclonal IgG antibody against TRPA1 (1:200; Novus, St. Charles, MO, United States). The specificity of the TRPA1 antibody purchased from the supplier had been previously validated (Yu et al., 2009). The washed sections were then incubated for 1 h at room temperature (25°C) with goat anti-rabbit IgG conjugated with horseradish peroxidase (HRP; 1:5000, Sigma). After being washed three times for 10 min with PBS, these sections were incubated with streptavidin conjugated with HRP at 37°C for 30 min. Visualization was performed using 3, 3’-diaminobenzidine tetrahydrochloride (DAB) for 3 min, and viewed under a light microscope at 400 × magnification. The slides were coded and analyzed by an observer without prior knowledge of the experimental procedures. For each of the five animals in every group, a section was randomly chosen, and five fields were randomly selected from each section. The Image Pro Plus 6.0 system (Media Cybernetics, MD, United States) was used to detect the integral optical density (IOD) of positively stained sections (brown staining) and identify TRPA1 in each field within an area of 25 μm^2^, including the four corners and central area. The total area of the region of interest (the epithelial layer) and the IOD was measured objectively. This software measurement, of the positively stained area containing TRPA1, was used to calculate positive immunostaining (IOD/entire positive area). The average quantitative value of five fields was used for statistical analysis. All data from each group were collected at the same time under the same conditions.

### RNA Extraction and Real-Time Fluorescent Quantitative PCR

Total RNA was extracted from the skin and tracheal tissues using the TRIzol reagent (Invitrogen, Waltham, MA, United States), according to the manufacturer’s instructions. Single-strand cDNA was synthesized for each sample with oligo (dT) as the primer, using a RevertAid First Strand cDNA Synthesis Kit (Invitrogen), following the manufacturer’s protocol. Total RNA (500 ng) was used in a 7500 Fast Real-Time PCR System (Applied Biosystems, Foster City, CA, United States) with FastStart Universal SYBR Green (Roche, Indianapolis, IN, United States) after cDNA synthesis. The PCR conditions were as follows: initial denaturation at 95°C (2 min), followed by 45 cycles of amplification at 95°C (5 s) and 60°C (10 s). Fold change of gene expression was calculated by the 2^– ΔΔCt^ method relative to the internal reference gene (GAPDH [glyceraldehyde 3-phosphate dehydrogenase]). Primers for the guinea pig genes were as follows:

TRPA1 – forward 5′-GCATGGCTCTGCATTTTGCT-3′,
reverse 3′-CCTGTGAAGCAGGGTCTCAT-5′.
GAPDH – forward 5′-ACCGTCAAGGCTGAGAATGG-3′,
reverse 3′-TGATTCACGCCCATCACGAA-5′.

### SP Detection in BALF by ELISA

The SP levels in the BALF were determined in duplicate using DuoSet ELISA kits with an assay range of 39.0–2500 pg/mL (R&D Systems, Minneapolis, MN, United States), according to the manufacturer’s instructions. The intra-assay and inter-assay variability of the measurement was 5% and 10%, respectively, across the range of concentrations measured. Every sample (50 μL) occupied three wells of the 96-well plate and the data obtained represented the average of three measurements.

### Western Blot

Tissues of the skin and trachea (100 mg each) were fully ground and lysed with 1 mL pyrolysis liquid. Protein concentrations (20 μL) were measured using a bicinchoninic acid assay kit (Thermo Fisher Scientific), according to the manufacturer’s protocol. Equal amounts of protein (30 μg) were subjected to 10% sodium dodecyl sulfate–polyacrylamide gel electrophoresis. Gels were run at 80 V for 30 min, followed by 120 V for 1 h, before being transferred onto a polyvinylidene fluoride membrane (Millipore, Burlington, MA, United States). After blocking with PBS containing 5% non-fat milk for 2 h at room temperature (25°C), the product was incubated overnight at 4°C, either with rabbit polyclonal anti-TRPA1 antibody (1:1000; Novus, St. Charles, MO, United States) or a GAPDH antibody (1:1000; Abcam, Cambridge, MA, United States). The membrane was washed three times for 5 min with 15 mL of Tris-buffered saline and Tween 20 and then incubated with HRP-conjugated goat anti-rabbit IgG antibody (1:2000; Sigma, Welwyn Garden City, United Kingdom) for 1 h at room temperature (25°C). After washing, 1 mL of a chemiluminescent substrate (Thermo Fisher Scientific) was added to the membrane. The signal was detected and quantified with an enhanced chemiluminescence system (Image Quant LAS-4000 MINI; GE Healthcare Bio-Sciences, Pittsburgh, PA, United States). For each of the five animals per group, the IOD of protein bands were detected using the Image Pro Plus system, and TRPA1 relative to the GAPDH density was determined.

### Immunofluorescence and Laser-Scanning Confocal Microscopy Analysis

Sections (4 μm thick) were cut from frozen skin and tracheal tissues on a freezing microtome (CM1520; Leica Biosystems, Shanghai, China), and kept at room temperature (25°C) for 30 min. The sections were then washed three times with PBS for 5 min, incubated for 5–10 min in 3% H_2_O_2_ to eliminate endogenous peroxidase activity, and then washed twice again with PBS for 5 min. Sections were then incubated for 1 h with a blocking solution (10% goat serum). They were then further incubated for 30 min with rabbit polyclonal anti-TRPA1 antibody (1:100, Novus) and mouse monoclonal anti-PGP9.5 antibody (1:200, Abcam), and then incubated with FITC-conjugated goat anti-rabbit/mouse IgG antibody (1:500; Proteintech, Rosemont, IL, United States) for 30 min at 37°C. Following nuclear staining with DAPI (1:1000; Thermo Fisher Scientific), the sections were observed and analyzed using a laser-scanning confocal microscope (Nikon Eclipse TI; Nikon, Tokyo, Japan). To ensure objectivity, six different microscopic fields were randomly selected along the bronchial and epithelial cross-sections of each sample by a researcher who did not know the experimental procedures. The size of all fields was similar, and the scale was 50 μm. The fluorescence intensity of green (PGP9.5) and red (TRPA1) in every image was detected by the Image Pro Plus 6.0 system (Media Cybernetics, MD, United States). The intensity of red fluorescence relative to green fluorescence represented a positive result. The data from each group were collected at the same time under the same conditions.

### Statistical Analysis

Data were presented as the mean ± SD, based on a normal distribution confirmed by the Shapiro–Wilk test. A one-way analysis of variance was used for multiple-comparison statistical analysis, followed by *post hoc* analysis of the Student–Newman–Keuls q-test between pairs of groups. Linear regression was applied for associations between variables, as well as the Pearson’s rank correlation test. Statistical analysis was performed (as described in each figure legend) using the GraphPad Prism 7.0 software (GraphPad Software, CA, United States), and a *p*-value of <0.05 was considered statistically significant.

## Results

### Cough Reactivity to Cinnamaldehyde After Cold Exposure

**FIGURE 1:**
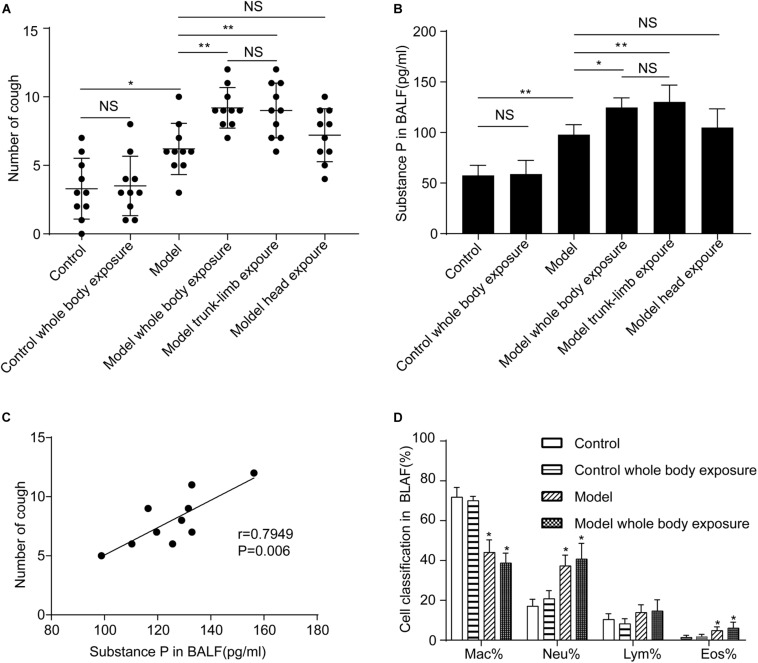
Effects of a cold environment on cough reactivity to inhaled cinnamaldehyde, and substance P levels or cell differential counts in the bronchoalveolar lavage fluid (BALF) of guinea pigs. **(A)** Repetitive inhalation of citric acid (model) markedly increased the number of cough events, as compared with the healthy group (control). Cough hyperreactivity was further aggravated when the whole body (model, whole-body exposure) or trunk-limbs (model, trunk-limb exposure), but not the head (model, head exposure) of guinea pigs with cough were exposed to a cold environment. In contrast, cough reactivity showed no change in healthy guinea pigs, following exposure to a cold environment (control, whole-body exposure), when they were completely exposed to a cold environment. **(B)** Substance P levels in the BALF of guinea pigs were increased in a similar pattern to the changes observed in cough reactivity, as presented in panel **(A)**. **(C)** A significant positive correlation was noted between substance P levels in BALF and cough reactivity in the guinea pigs with cough (model). **(D)** Repetitive inhalation of citric acid significantly increased the percentages of neutrophils and eosinophils in the BALF. Results are presented as the mean ± SD (*n* = 10; *p < 0.05, **p < 0.01),

Repeated inhalation of citric acid markedly increased cough reactivity of guinea pigs to inhaled cinnamaldehyde, as compared with the control (3.3 ± 0.7 coughs vs. 6.2 ± 0.6 coughs, *n* = 10, *p* < 0.05). Cough hyperreactivity was further aggravated when guinea pigs with a cough were subjected to whole-body exposure to a cold environment (6.2 ± 0.6 coughs vs. 9.2 ± 0.5 coughs, *n* = 10, *p* < 0.05). In contrast, exposure to a cold environment induced no increased cough in the control group (*p* > 0.05) (Figure 1A).

We evaluated changes in cough reactivity in guinea pigs with cough following their exposure to a cold environment, using three different modes. As expected, trunk-limb exposure to the cold environment induced higher cough reactivity (6.2 ± 0.6 coughs vs. 9.0 ± 0.6 coughs, *n* = 10, *p* < 0.05), which was comparable with that of whole-body exposure (9.2 ± 0.5 coughs vs. 9.0 ± 0.6 coughs, *n* = 10, *p* > 0.05) (Figure 1A). However, head exposure to the cold environment did not promote cough reactivity (6.2 ± 0.6 coughs vs. 7.2 ± 0.6 coughs, *n* = 10, *p* > 0.05).

### Effects of a Cold Environment on SP Levels and Cell Differential Counts in BALF

The repeated inhalation of citric acid resulted in a marked increase of SP in the BALF (*p* < 0.05), as measured by ELISA. No apparent differences were observed between guinea pigs subjected to repeated inhalation of saline, which were also exposed to the cold environment, and those which were not (*p* > 0.05) (Figure 1B). However, higher SP levels were detected in guinea pigs with cough, which were subjected to either whole-body exposure or trunk-limb exposure to the cold environment, but not head exposure (Figure 1B). A significant positive linear correlation was noted between SP levels in the BALF and cough in guinea pigs (Figure 1C). Repeated inhalation of citric acid significantly increased the percentages of neutrophils and eosinophils in the BALF (Figure 1D).

### Effects of a Cold Environment on TRPA1 Expression

**FIGURE 2:**
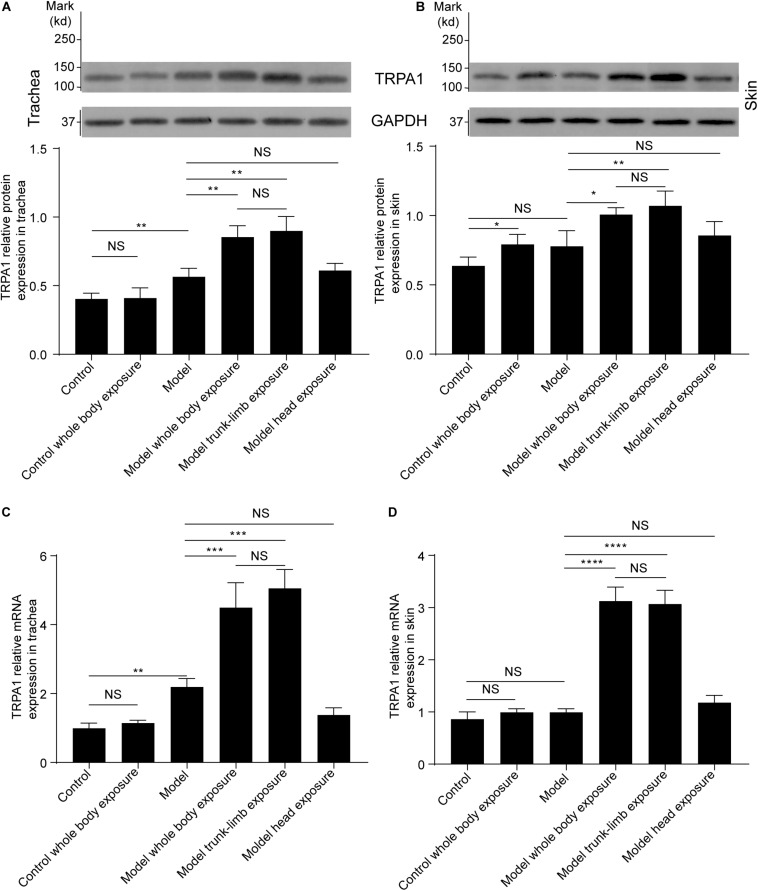
TRPA1 protein and mRNA expression in the trachea and skin tissues of guinea pigs, as detected by western blot and RT-PCR, respectively. **(A)** Cold exposure increased expression of the TRPA1 protein in the trachea of guinea pigs with cough (model), but not in healthy guinea pigs (control). Similar enhancement of TRPA1 overexpression was observed in the guinea pigs with cough, following exposure of the whole body (model, whole-body exposure) and trunk-limbs (model, trunk-limb exposure), but not the head (model, head exposure), to a cold environment. **(B)** Cold exposure increased the expression of TRPA1 protein in the skin of healthy guinea pigs (control, whole-body exposure), and guinea pigs with cough (model), following exposure of the whole body (model, whole-body exposure) and trunk-limbs (model, trunk-limb exposure), but not the head (model, head exposure), to a cold environment. **(C)** TRPA1 mRNA expression in the trachea was similar to TRPA1 protein expression among the different groups of guinea pigs, as presented in panel **(A)**. **(D)** The increase in TRPA1 mRNA expression in the skin was remarkable only in the guinea pigs with cough, following exposure of the whole body (model, whole-body exposure) and trunk-limbs (model, trunk-limb exposure) to a cold environment. Results are presented as the mean ± SD (*n* = 5; *p < 0.05, **p < 0.01, ***p < 0.001, ****p < 0.0001).

**FIGURE 3:**
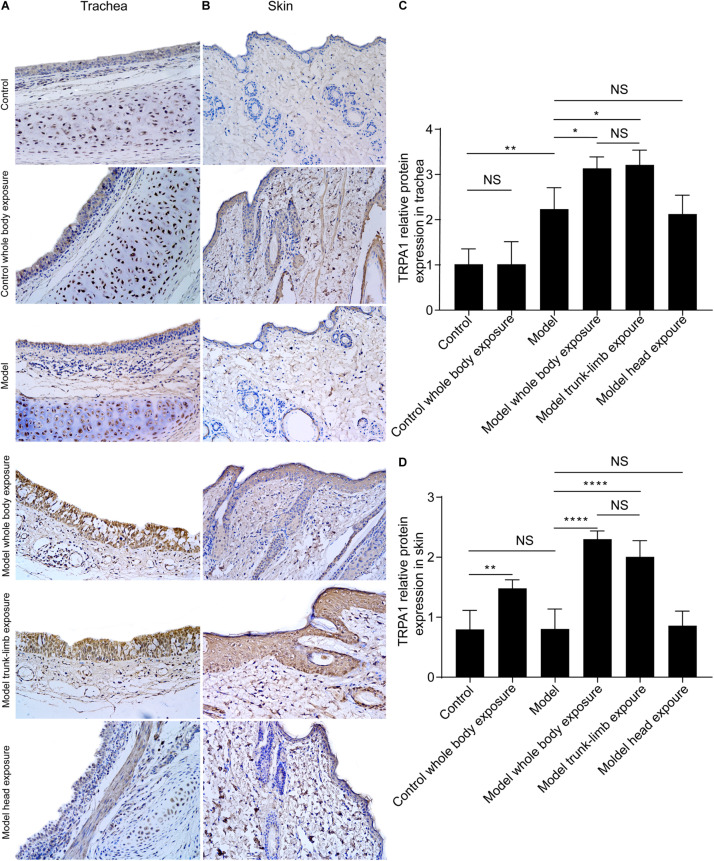
TRPA1 expression and distribution in the trachea and skin tissues of guinea pigs, as detected by immunohistochemistry. **(A)** TRPA1 expression and distribution (shown as brown staining) in the trachea of different groups of guinea pigs. **(B)** TRPA1 expression and distribution (shown as brown staining) in the skin of different groups of guinea pigs. **(C)** Digital quantitative presentation of TRPA1 expression in the trachea of different groups of guinea pigs, demonstrating that TRPA1 overexpression in the trachea was induced by the repetitive inhalation of citric acid (model) and further enhanced, following exposure of the whole body (model, whole-body exposure) and trunk-limbs (model, trunk-limb exposure), but not the head (model head exposure), to a cold environment. **(D)** Digital quantitative presentation of TRPA1 expression in the skin of different groups of guinea pigs, showing that TRPA1 overexpression in the skin was induced in healthy guinea pigs (whole-body control) and further elevated, following exposure of the whole body (model, whole-body exposure) and trunk-limbs (model, trunk-limb exposure), but not the head (model, head exposure), to a cold environment. The original magnification was 400× for panels **(A,B)**, and the quantitative values (mean ± SD) of stained epithelia are presented for panels **(C,D)** (*n* = 5; *p < 0.05, **p < 0.01, ****p < 0.0001).

As evidenced by western blot analysis, repeated inhalation of citric acid significantly increased expression of the TRPA1 protein in the trachea (Figures 2A, 3A,C), but not in the skin (Figures 2B, 3B,D). Cold exposure alone increased the expression of TRPA1 in the skin (Figures 2B, 3B,D), but not in the trachea (Figures 2A, 3A). In particular, higher expression of the TRPA1 protein was observed in the trachea (Figures 2A, 3A,C) of guinea pigs with cough that had been subjected to either whole-body exposure or trunk-limb exposure to the cold environment, but not head exposure. These observations were further verified by real-time quantitative PCR, which detected similar trends in mRNA overexpression of TRPA1 in the skin and trachea of guinea pigs with a cough that were subjected to whole-body exposure or trunk-limb exposure to the cold environment, but not head exposure (Figures 2C,D).

### Effects of TRPA1 Antagonist on Cough Reactivity and TRPA1 Expression

**FIGURE 4:**
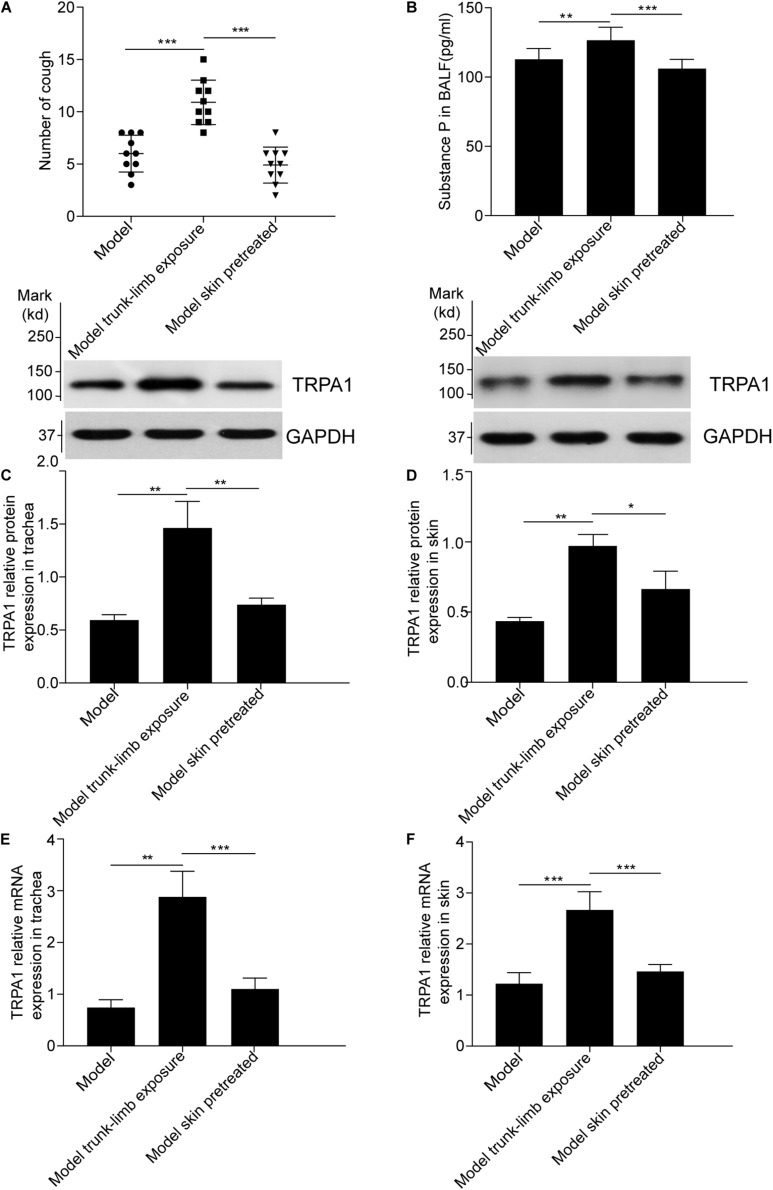
Blockade of cough hyperreactivity to inhaled cinnamaldehyde, increased substance P levels, and TRPA1 overexpression following pretreatment of the skin with HC-030031, a TRPA1 selective antagonist, in guinea pigs with cough. **(A)** The skin of model guinea pigs were pretreated with HC-030031, then exposed to cold environment (model skin treated), the cough reactivity showed decreased significantly, as compared with the model trunk-limbs cold exposure group. **(B).** Pretreatment of the skin with HC-030031 completely blocked the increase in substance P levels in the bronchoalveolar lavage fluid (BALF) of guinea pigs with cough, the trunk-limbs of which were exposed to a cold environment. **(C–F)** The increase in TRPA1 protein levels and mRNA overexpression in the trachea and skin were inhibited, following pretreatment of the skin with HC-030031, in guinea pigs with cough, the trunk-limbs of which were exposed to a cold environment, as detected by western blot and RT-PCR. Data are expressed as the mean ± SD (*n* = 10; *p < 0.05, **p < 0.01, ***p < 0.001).

**FIGURE 5:**
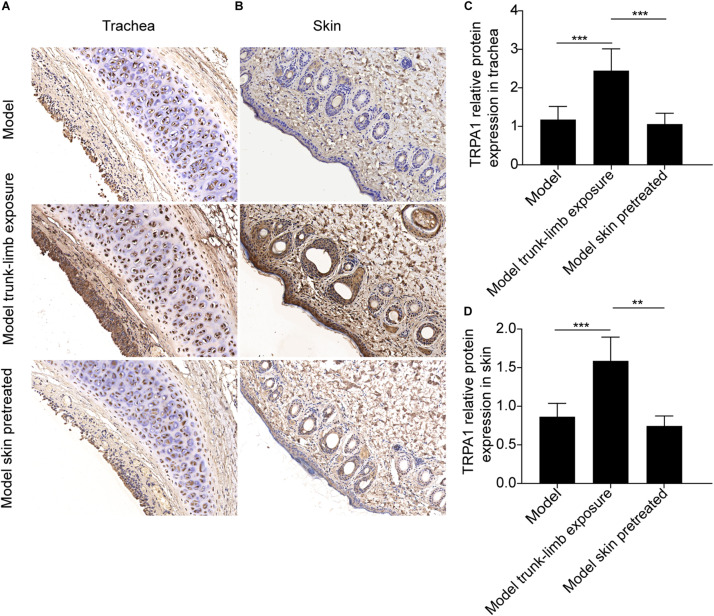
Pretreatment of the skin with HC-030031, a TRPA1 selective antagonist, altered TRPA1 expression and distribution (as detected by immunohistochemistry) in the trachea and skin tissues of guinea pigs with cough, the trunk-limbs of which were exposed to a cold environment. **(A)** TRPA1 expression and distribution (shown as brown staining) in the trachea of guinea pigs with cough (model); guinea pigs with cough, the trunk-limbs of which were exposed to a cold environment (model, cold exposure); and guinea pigs with cough, the trunk-limbs of which were exposed to a cold environment, but also had HC-030031-pretreated skin (model, skin pretreated). **(B)** TRPA1 expression and distribution (shown as brown staining) in the skin of different groups of guinea pigs with cough. **(C)** Digital quantitative presentation of TRPA1 expression in the trachea of different groups of guinea pigs, demonstrating that pretreatment of the skin with HC-030031 abolished TRPA1 overexpression in the trachea of guinea pigs with cough that were exposed to a cold environment. **(D)** Digital quantitative presentation of TRPA1 expression in the skin of different groups of guinea pigs, showing that pretreatment of the skin with HC-030031 blocked TRPA1 overexpression in the skin of guinea pigs with cough, the trunk-limbs of which were exposed to a cold environment. The original magnification was 400 × for panels **(A,B)**, and the quantitative values (mean ± SD) of stained epithelia are presented for panels **(C,D)** (*n* = 5; **p < 0.01, ***p < 0.001).

To confirm the role of the skin TRPA1 signaling pathway in cough reflex hyperreactivity aggravated by cold environments, we used HC-030031, a selective TRPA1 antagonist, to pretreat the guinea pigs with cough. The trunk-limbs of those animals were immersed in 1.6 mM HC-030031 solution before exposure to the cold environment. As shown in Figure 4A, blockade of skin TRPA1 following HC-030031 immersion inhibited cough reflex hyperreactivity and TRPA1 overexpression (Figures 4C–F, 5) aggravated by exposure to a cold environment, and abolished the increase in SP levels in the BALF (Figure 4B).

### TRPA1 Expression on Nerve Fibers in the Skin and Trachea

**FIGURE 6:**
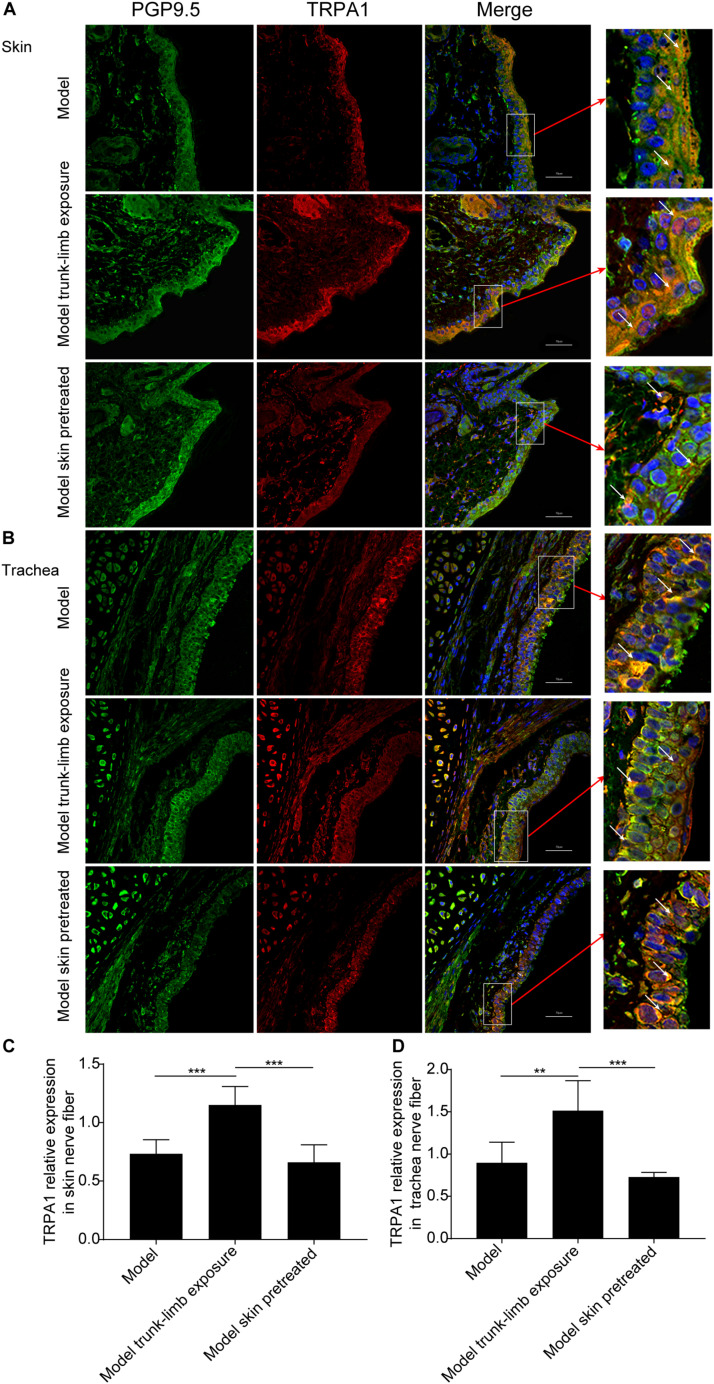
TRPA1 expression on nerve fibers in the trachea and skin of guinea pigs, as detected by immunofluorescence and confocal microscopy. **(A,B)** Representative TRPA1 expression (shown as red fluorescence) and PGP9.5 positive nerve fiber distribution (shown as green fluorescence) in the skin **(A)** and trachea **(B)** of guinea pigs with cough (model); guinea pigs with cough, the trunk-limbs of which were exposed to a cold environment (model, trunk-limb cold exposure); and guinea pigs with cough that were exposed to a cold environment, but with HC-030031-pretreated skin (model, skin pretreated). Positive fluorescence (white arrow) was primarily distributed on the epithelia. **(C,D)** Digital quantitative presentation of TRPA1 expression on the nerve fibers in the skin **(C)** and trachea **(D)** of different groups of guinea pigs. Results are presented as the mean ± SD (*n* = 5; **p < 0.01, ***p < 0.001).

We further verified whether a cold environment can promote TRPA1 expression on sensory afferents in the skin, and then excite TRPA1 on sensory afferents in the airways by cross-talk. We achieved this by detecting TRPA1 expression on nerve fibers in the skin and trachea using immunofluorescence and confocal microscopy. Nerve fibers in the skin and trachea were labeled with the selectivity nerve fiber fluorescent antibody, PGP9.5 (green fluorescence), and the expression of TRPA1 was shown as red fluorescence (Figure 6). The ratio between the intensity of red and green fluorescence was significantly increased after exposure of the guinea pigs with cough to a cold environment. In addition, the enhanced fluorescence was mainly distributed in the dermis of the skin and mucosa of the trachea (Figure 6). Moreover, pretreatment with HC-030031 blocked the overexpression of TRPA1 on nerve fibers in the skin and trachea (Figure 6).

## Discussion

This study demonstrated that exposure to a cold environment can aggravate cough hyperreactivity to inhaled cinnamaldehyde in guinea pigs with cough induced by repeated inhalation of citric acid. Exaggerated cough hyperreactivity occurred only in guinea pigs with a cough that were subjected to either whole-body or trunk-limb exposure to the cold environment. However, the aggravated cough hyperreactivity was reduced by pretreatment of the skin with the selective TRPA1 inhibitor, HC-030031. The SP levels in the BALF, and TRPA1 expression in the tracheal mucosa and skin were upregulated in guinea pigs with a cough that had been subjected to either whole-body or trunk-limb exposure to the cold environment, rather than those subjected to only head exposure. This trend was abolished after pretreatment of the skin with HC-030031. TRPA1 overexpression and clustered distribution were localized to nerve fibers in the tracheal mucosa and skin, as identified by immunofluorescence and laser-scanning confocal microscopy analyses.

Understanding of chronic cough is hindered largely by the lack of appropriate animal models. Although acute cough models are useful for the investigation of cough pathogenesis and antitussive effects, they cannot reflect the actual pathophysiology of chronic cough (Wex and Bouyssou, 2015), and therefore are not representative of, or applicable to human diseases. This study used repeated inhalation of citric acid to establish the prolonged cough model in guinea pigs because citric acid is a classic protussive agent that induces cough by stimulating both C-fiber and A-fiber terminals (but primarily C-fiber afferents) (Tanaka and Maruyama, 2005). Cough hypersensitivity is evident in the guinea pig after the repetitive inhalation of citric acid and may be associated with airway mechanical injury, neurogenic inflammation, and remodeling – as demonstrated by our team (Xu et al., 2018) and another group (Nakaji et al., 2016; Cui et al., 2019). This is supported by the increased SP levels in the BALF. We also observed a mild increase in the percentage of eosinophils in BALF after citric acid inhalation, but not after cold exposure. The airway eosinophilia was comparable to that observed in our previous study (Xu et al., 2018) and was possibly related to epithelial injury in the airway induced by the cough itself and bronchoconstriction during the repeated inhalation of citric acid (Fang et al., 2019). Such phenomena can be more representative of, or applicable to, the disease process of chronic cough in humans. Moreover, the repeated inhalation of citric acid triggered excessive cough, following exposure to the cold environment, and reflected a characteristic of the cough hypersensitivity syndrome, particularly refractory chronic cough in humans (Chung, 2011; Morice, 2013). This study thus confirmed that a cold environment, an innocuous stimulus unassociated with cough, can affect cough exacerbation, under the conditions of cough hypersensitivity induced by repeated irritation of the airways. Nevertheless, cold exposure had no effect on cough reactivity in healthy guinea pigs, which is consistent with the findings of a previous study by Yoshihara et al. (1996).

In patients with respiratory diseases cough is induced and aggravated by exposure to a cold environment (Cho et al., 2003; Huang et al., 2015; Hyrkas et al., 2016; Kanemitsu et al., 2016). The phenomenon of cough exacerbation in cold seasons and environments is believed to be related to the activation of cough receptors elicited by the inhalation of cold air (Koskela, 2007). In contrast, our results indicate that the inhalation of cold air simulated by head exposure to the cold environment did not contribute to cough hyperreactivity. Although the direct inhalation of cold air into the lower airways can provoke a cough (Banner et al., 1985), the unique structure of the upper respiratory tract of the human body endows the airways with the physiological function to warm and humidify inhaled air, meaning cold air does not usually enter the lower airways. This helps to keep the internal environment of the human body relatively stable. This phenomenon was confirmed by our results, which showed that inhaled cold air was warmed up to 32–33°C in the upper airways and trachea. Consistent with our findings, Plevkova et al. (2013) showed that transnasal inhalation of cold air inhibits the cough reflex in guinea pigs, by gating the cold-sensitive cation channel TRPM8 protein expressed on trigeminal nasal afferent neurons. The frequent cough of athletes during winter training can be attributed to open-mouth breathing (Turmel et al., 2012). Furthermore, cough exacerbation in patients with severe chronic obstructive pulmonary disease, or those affected by asthma attacks on exposure to cold environments (Huang et al., 2015; Kanemitsu et al., 2016), may be caused by a switch in breathing pattern from the transnasal mode and a bypass of the protective mechanisms mentioned above (Turmel et al., 2012). However, in most instances, there is little possibility that cold air directly stimulates the cold-sensitive cough receptors innervating the lower airways in patients with chronic bronchitis and chronic cough. The present study showed that under pre-existing conditions of increased cough sensitivity, cough reactivity was further exaggerated by cold exposure. This may be related to the direct activation of cold receptors in the skin that sense cold stimuli.

When considering cough exacerbation during exposure to a cold environment, one can argue that the skin of the trunk and limbs would be less stimulated by cold air than that of the head and face, as the human torso is generally covered by warm clothing and maintained at a higher temperature than that of the head and face. In the present study, we exposed the head and face of the guinea pigs to simulate the inhalation of cold air in humans, as the sole transnasal inhalation of cold air was impossible in conscious guinea pigs. Therefore, our model imitated transnasal breathing in humans but did not fully replicate face and head exposure in humans. Moreover, the skin on the head and face of humans can likely tolerate cold air owing to our long-term adaptation to the environment. A person’s choice of clothing is also a significant and variable factor in maintaining sufficient body warmth in patients with chronic cough. In addition to the fact that transnasal inhalation of cold air inhibited the cough reflex in guinea pigs (Plevkova et al., 2013), Koskela and Tukiainen (1995) have demonstrated that bronchoconstriction induced by cold climates in asthmatic patients was mediated by cooling the skin, but not transnasal inhalation of cold air. They also observed that one asthmatic patient developed a severe cough after whole-body exposure to subfreezing air, but not after transnasal inhalation of subfreezing air (Koskela and Tukiainen, 1995). Therefore, our findings may explain cough exacerbation in patients with a chronic cough during seasonal changes and cold temperatures, including entry to an air-conditioned room.

The primary input for peripheral temperature comes from sensory afferents that detect the temperature of the body. Most of these sensory neurons have cell bodies located in the trigeminal ganglion (for innervation of the head and face) and dorsal root ganglia (for innervation of the rest of the body). The axons of these sensory neurons extend outward to measure the temperature of key thermoregulatory tissues, such as the skin and viscera (Tan and Knight, 2018). Both TRPA1 and TRPM8, expressed on sensory afferents, are triggered by cold temperatures. The TRPA1 protein is responsible for sensing noxious, painful, cold stimuli and TRPM8 is responsible for perceiving innocuous cool temperatures (Patapoutian et al., 2003; Ferrer-Montiel et al., 2012). At present, TRPM8 is considered the primary peripheral cold sensor in the skin, and its expression is required for cold perception (Tan and Knight, 2018). However, TRPA1’s contribution to cold perception *in vivo* is still a matter of debate. Nevertheless, much evidence shows that TRPA1 is a cold sensor in the skin. Munns et al. (2007) showed no correlation between TRPA1 and cold temperature in dorsal root ganglion neurons isolated and cultured from mice. Other researchers demonstrated that dorsal root ganglion neurons can be cold-sensitive via TRPA1 activation (Dhaka et al., 2006). Recently, Winter et al. (2017) illustrated the considerable contribution of TRPA1 to both innocuous cold sensing and noxious cold sensing in the somatosensory system. As the activation of cold sensing TRPM8 causes inhibition of the cough reflex, we speculate that TRPA1 in the skin may be a key cold receptor associated with further cough exaggeration, following exposure to a cold environment. Pan et al. (2018) reported that TRPA1 is essential for cutaneous vasoconstriction, following cold exposure in mice. Taking this into account, we examined the effects of immersion in the selective TRPA1 antagonist, HC-030031, which was designed to block TRPA1 activation in the skin of trunk-limbs, but not in the airways of the guinea pigs. We found that pretreatment with HC-030031 almost completely inhibited cough hyperreactivity induced by cold exposure. This suggested that the TRPA1 signaling pathway in the skin effected cough exacerbation, following exposure to a cold environment. The alternative explanation was the direct action of HC-030031 on the TRPA1 of the airway, due to its absorption through the skin and high systemic bioavailability. However, HC-030031 is not absorbed into the blood through the skin, because it cannot be dissolved in water or saline. Our results again suggest that cold-induced cough exacerbation was mediated through the dorsal root ganglia, in addition to the trigeminal ganglion. We infer this because neither cold exposure of the skin on the head and face of guinea pigs nor the inhalation of cold air was able to aggravate cough hyperreactivity.

The TRPA1 protein is widely distributed on nasal trigeminal afferents innervating the upper airways (Plevkova et al., 2013). In contrast, only 15–16% of pulmonary vagal sensory fibers express TRPA1 in rats (Xing et al., 2008; Zhou et al., 2011). The abundance of TRPA1 on sensory endings in the upper respiratory tract is consistent with the high frequency of exposure of the nose to cold air. Similarly, the paucity of TRPA1 in the lower airways reflects an evolutionary adaptation to warmer environments within the lungs. In the present study, repetitive inhalation of citric acid only induced TRPA1 overexpression of the sensory endings and tissues in the trachea of guinea pigs with cough via local irritation of the airways. Similarly, cold exposure merely increased TRPA1 expression in the stimulated skin of control guinea pigs. When the whole bodies or trunk-limbs of guinea pigs with cough were exposed to the cold environment, cough hyperreactivity was further enhanced along with increased expression of TRPA1 on the sensory endings and tissues of the trachea and increased SP levels in the BALF. These trends were abolished by pretreatment of the skin of the trunk-limbs with the selective TRPA1 antagonist. These results suggest that the establishment of cross-talk of TRPA1 on sensory fibers between the lower airways and skin is synergistically promoted by cough reflex hypersensitivity and cold environments, and its association with cough hyperreactivity is aggravated by a cold environment. Previous studies have demonstrated that patients with allergic dermatitis, without respiratory symptoms or localized scleroderma, present with subclinical neurogenic inflammation in the lower respiratory tract and cough hypersensitivity to inhaled capsaicin (Pecova et al., 2003a, b). Therefore, inflammatory stimulation of the skin can trigger the development of airway pathophysiology and activate cough receptors. This theory is supported by our findings that the TRPA1 signaling pathway in the skin links airway neurogenic inflammation, induced by activation of the sensory fibers expressing TRPA1 in the airways, with the consequent release of neuropeptides. This release precipitated cough hyperreactivity induced by the cold environment in guinea pigs with cough.

As cough hypersensitivity syndrome in humans can be affected by a wide range of stimuli, we expected the guinea pigs with cough to present exacerbated cough reactivity to various tussive agents, including TRPV1 and TRPA1. Therefore, it was remarkable that exaggerated cough hyperreactivity was only induced by the TRPA1 agonist, cinnamaldehyde, but not by the TRPV1 agonist, capsaicin, following exposure of the guinea pigs with cough to a cold environment. A previous report has shown that the heterogeneity of cough hypersensitivity, mediated by TRPV1 and TRPA1, is common in patients with chronic refractory cough (Long et al., 2019). Morice et al. (2019) demonstrated that a P2X3 receptor antagonist decreased cough sensitivity to inhaled ATP, but not to inhaled citric acid and capsaicin, in patients with chronic cough. This suggests the existence of distinct pathways affecting the cough reflex. Our results showed that repeated citric acid challenge increases cough responsiveness to inhaled cinnamaldehyde and capsaicin. These findings are consistent with those of a previous study by Xu et al. (2018) and could be attributed to the simultaneous activation of TRPA1 and TRPV1 co-expressed in vagal C-fibers in the airways (Grace et al., 2014). However, exposure to a cold environment only further increased cough responsiveness to cinnamaldehyde, but not capsaicin. This highlights a unique pathway involving TRPA1, but not TRPV1, because of its function in sensing differences in temperature. Yoshihara et al. (1996) have shown that skin stimulation with cold water inhibits capsaicin-induced cough in normal guinea pigs. Therefore, we believe that cough hyperresponsiveness to a cold environment is a unique pathway involving TRPA1, but not TRPV1, owing to its function in sensing a difference in temperature.

The mechanism by which TRPA1 detects cold signals on the skin to aggravate cough hyperreactivity in guinea pigs with cough remains unclear. Several mechanisms may be proposed. The activation of TRPA1 on skin sensory nerve endings by cold environments might stimulate nearby mast cells to release inflammatory mediators, such as histamine, into the blood and thereby excite cough receptors in the airways. This theory is supported by the fact that TRPA1 is extensively distributed on skin sensory nerve endings (Winter et al., 2017) and a structural connection exists between mast cells in the skin and afferent sensory fibers (Jarvikallio et al., 2003). However, this theory is unlikely, considering the dilution and clearance of inflammatory mediators with a protussive effect in circulating blood. The direct cross-talk of TRPA1 on the sensory afferents between the skin and airways through axonal reflexes may be possible but is also unlikely, as no evidence exists to show the anatomical link between the nerve fibers in both locations. Moreover, the exact mechanism underlying our findings may involve the cough center. At the level of the brainstem, the nucleus tractus solitarius associated with the cough center is not only regulated by the higher cortical center; it is also closely related to other functional centers of the body (Widdicombe et al., 2011). The paraventricular nucleus, a center for regulating body temperature, receives afferent terminations from the nucleus tractus solitarius, emits efferent projections to the cough center, and transmits synaptic signals through SP and its receptor, neurokinin 1 (El-Hashim et al., 2018). Afferent impulses from the nose, mouth, throat, esophagus, and skin can converge in the cough center, and modify cough sensitivity by affecting the interactions among different types of receptors and the functional relationships between various centers (Widdicombe et al., 2011). Therefore, we believe the more likely notion that when the nucleus tractus solitarius was sensitized, following the increased peripheral protussive input induced by repeated inhalation of citric acid, TRPA1 expression on the afferent nerves in the skin was promoted by the cold environment. This may lead to the transmission of cold signals to the central paraventricular nucleus, where information is integrated and delivered to the nucleus tractus solitarius through the efferent pathway to further sensitize and excite the cough center. This process leads to the amplification of incoming protussive signals from the airways and aggravates cough hyperreactivity (Canning, 2009).

The current study demonstrates that under the conditions of cough hypersensitivity and exaggerated cough hyperreactivity induced by a cold environment, are associated with the activation of the cold-sensing TRPA1 signaling pathway in the skin, rather than the inhalation of cold air. These findings provide evidence that may help develop more effective strategies in preventing chronic cough, such as maintaining body warmth by wearing sufficient clothing in the winter. Moreover, TRPA1 may become a potential therapeutic target in the management of chronic cough, especially in preventing cough exacerbation in cold weather. However, future studies are needed to confirm the relevant mechanisms at the level of the central nervous system.

## Data Availability Statement

The datasets generated for this study are available on request to the corresponding author.

## Ethics Statement

The animal study was reviewed and approved by Ethical Committee for Care and Use of Laboratory Animals at Tongji Hospital.

## Author Contributions

ZQ was in charge of the conception and design of the research. RD, TZ, and WW performed the experiments. RD and MZ analyzed the data and interpreted the results of the experiments. RD and TZ drafted the manuscript. RD, QC, and XX prepared the figures. ZQ, WW, and LY reviewed and revised the manuscript. All authors approved the final version of the manuscript.

## Conflict of Interest

The authors declare that the research was conducted in the absence of any commercial or financial relationships that could be construed as a potential conflict of interest.
