# Supplementary figures and images for: A Cold Environment Aggravates Cough Hyperreactivity in Guinea Pigs With Cough by Activating the TRPA1 Signaling Pathway in Skin
Source: Front Physiol. 2020 Aug 27;11:833. doi: 10.3389/fphys.2020.00833 (PMC7481366; doi:10.3389/fphys.2020.00833)

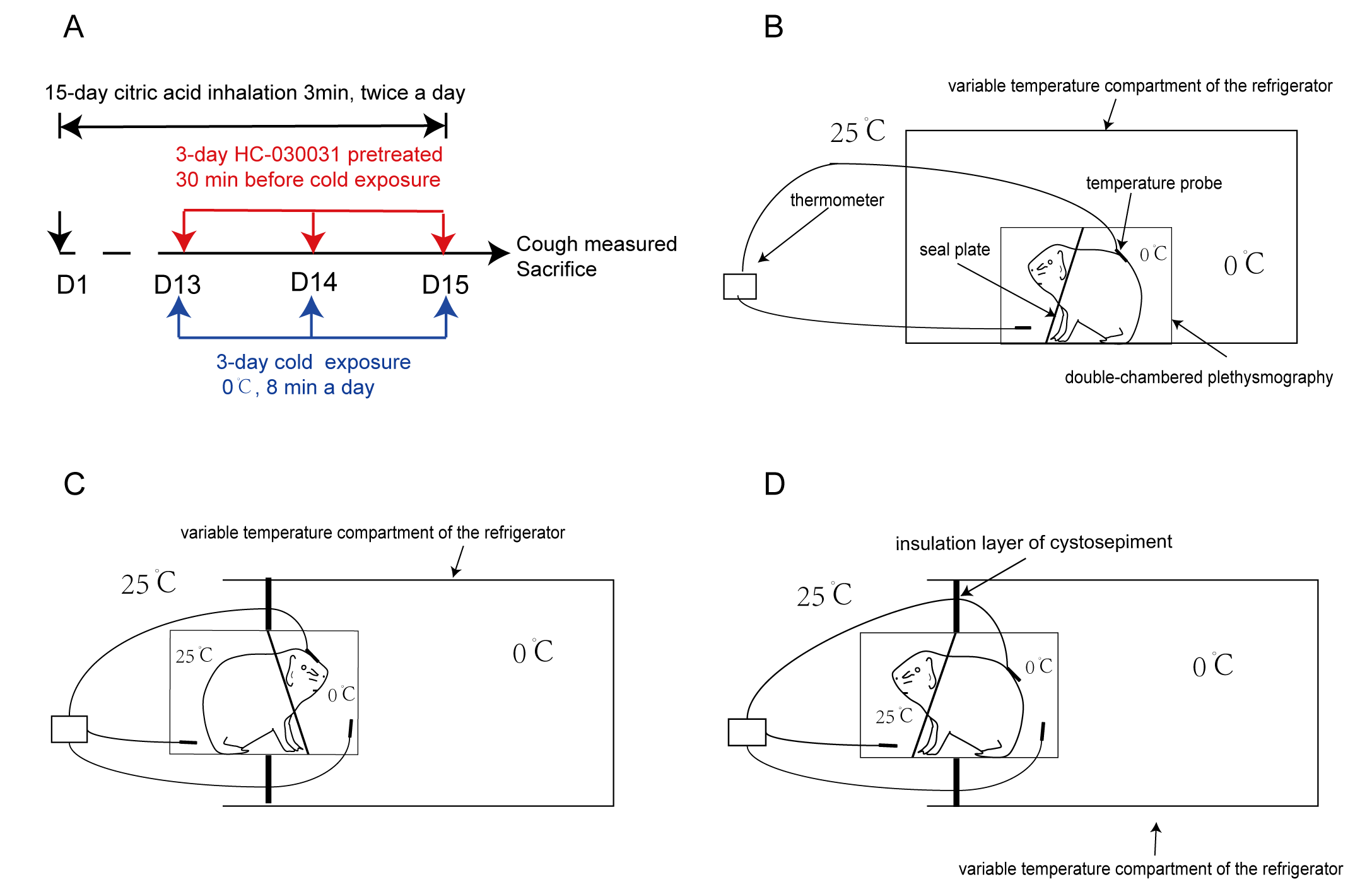

Supplement: Supplementary file 1 [file Image_1.TIF]

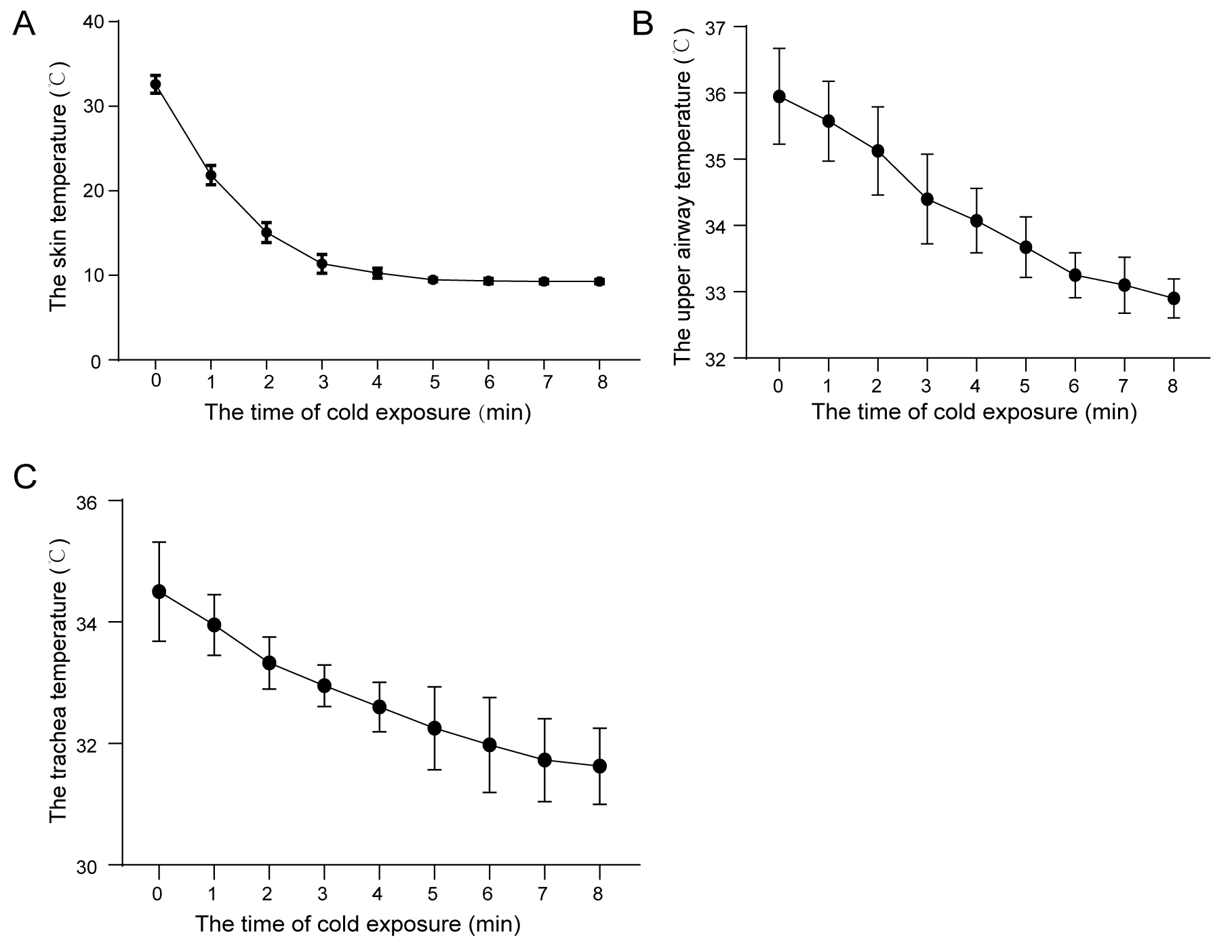

Supplement: Supplementary file 2 [file Image_2.TIF]

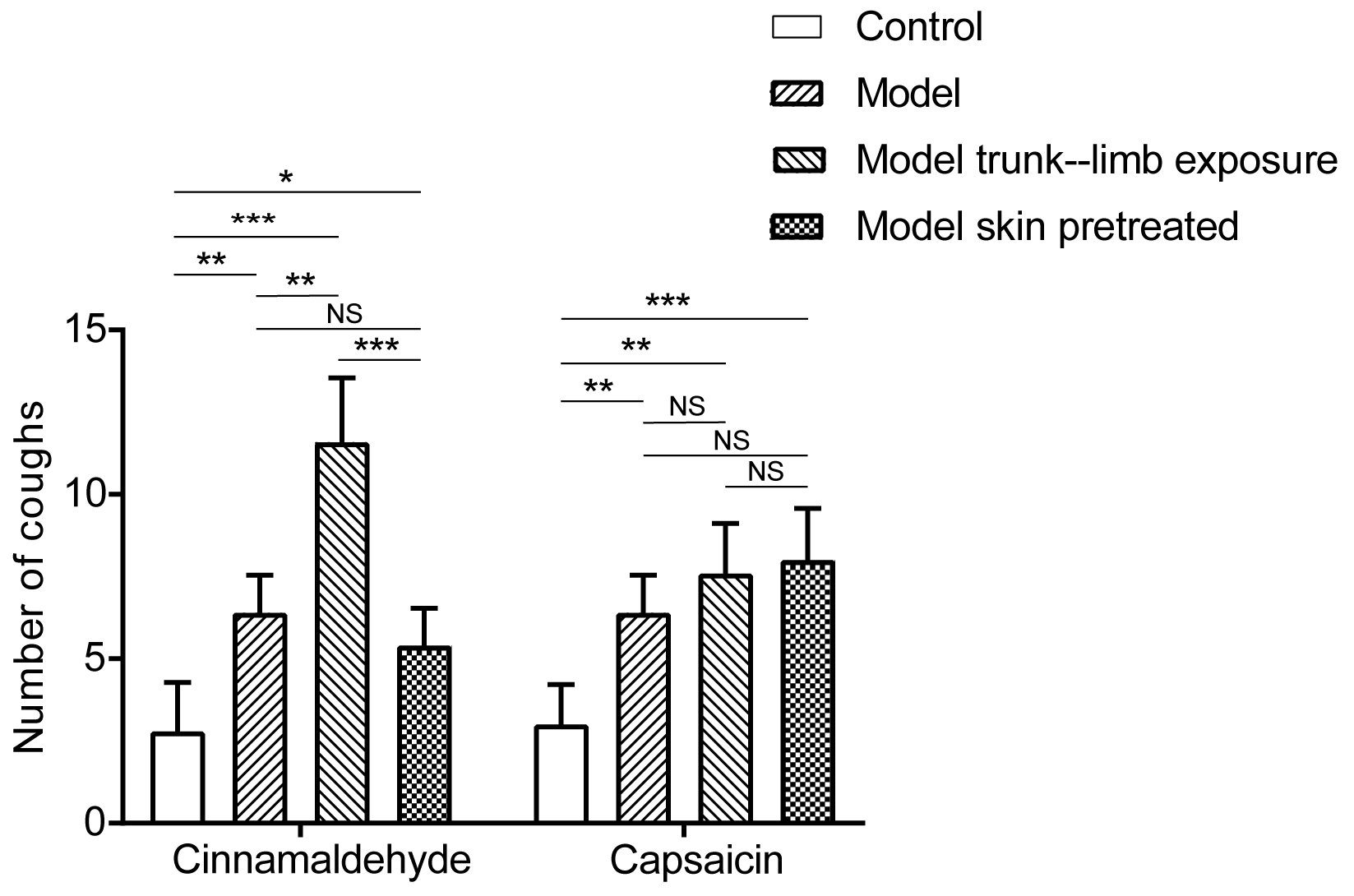

Supplement: Supplementary file 3 [file Image_3.TIF]

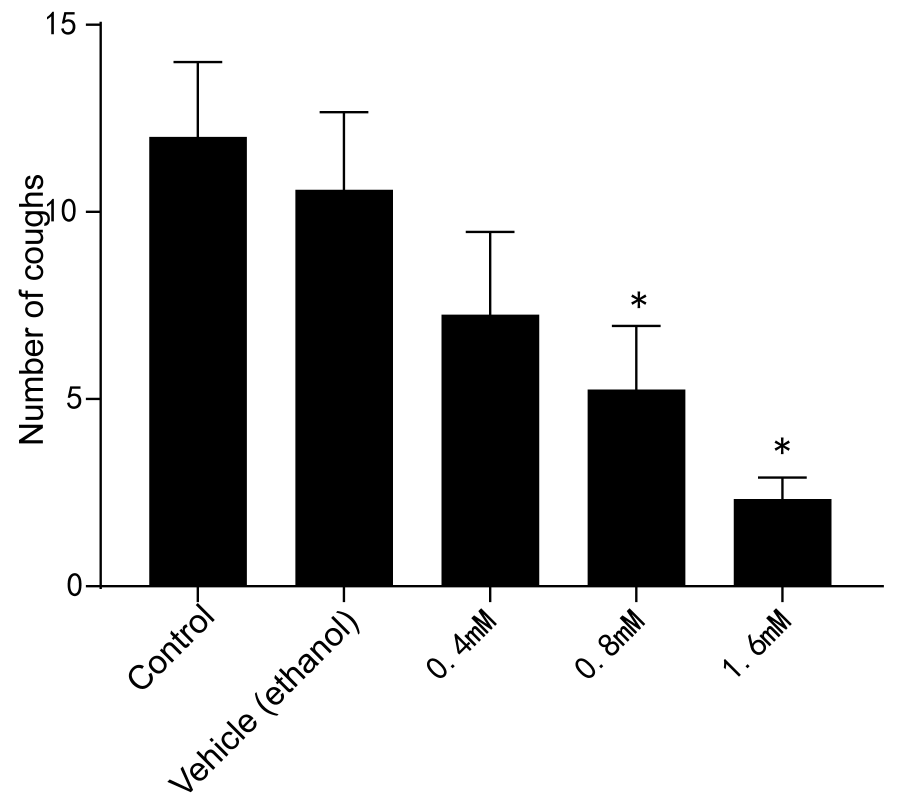

Supplement: Supplementary file 4 [file Image_4.TIF]

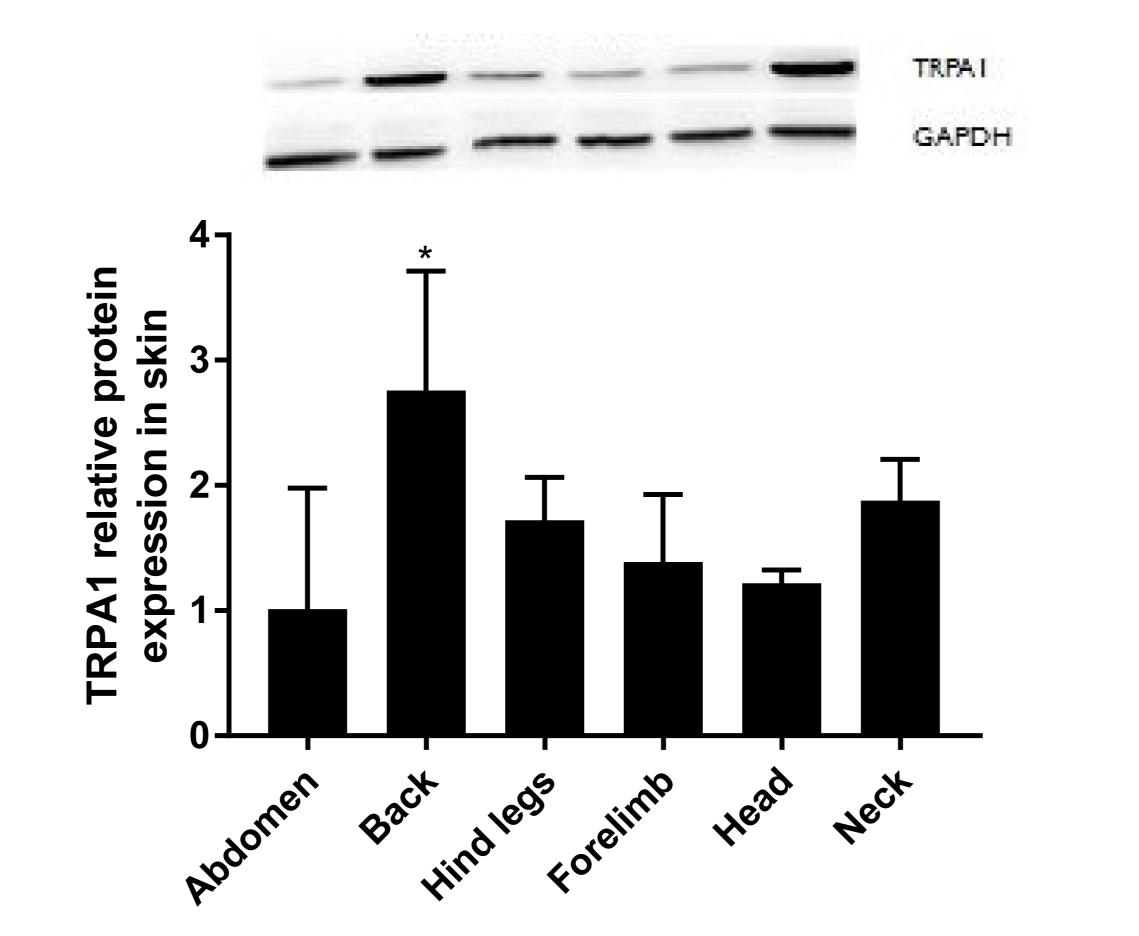

Supplement: Supplementary file 5 [file Image_5.TIF]
