# Supplementary material for: A Cold Environment Aggravates Cough Hyperreactivity in Guinea Pigs With Cough by Activating the TRPA1 Signaling Pathway in Skin
Source: Front Physiol. 2020 Aug 27;11:833. doi: 10.3389/fphys.2020.00833 (PMC7481366; doi:10.3389/fphys.2020.00833)
Supplement: Supplementary file 6 [file Table_1.doc]

Supplementary Material

# Supplementary Methods

*Detection of skin, upper airways and* trachea temperature during cold exposure (Figure S2)

# For the temperature measurement in upper airway and trachea, the guinea pigs were anesthetized by an intraperitoneal injection of 15mg/kg pentobarbital sodium and were placed in the supine position. After a probe (0.1mm) of the thermometer (DT-1, Jingchuang Electronics Co. Ltd, Jiangsu, China) was inserted into the mouth and trachea of the guinea pigs, the animals were exposed to cold environment 11established in the variable temperature compartment of a refrigerator (Haier, China) as described in the text, and the changes in the temperature of the upper airway and trachea were monitored in real time and recorded every min during the 8 min course of cold exposure to 0ºC. In the case of the detection of the skin temperature following the exposure to cold environment, the probe of the thermometer was stick on the back skin of the [conscious](https://fanyi.baidu.com/#en/zh/conscious) guinea pigs, and the changes in the temperature was monitored and recorded according to the same procedure as described above.

# Supplementary Figure Legends

Figure S1. Schematic of the study and different parts of the cold exposure apparatus for guinea pigs. A: Schematic of the study. B. Whole-body exposure to a cold environment. C. Head exposure to a cold environment. D. Trunk-limb exposure to a cold environment.

Figure S2. Changes observed in the guinea pigs against time of exposure to a cold environment (0°C). A. Temperature of the skin. B. Temperature in the upper airways. C. Temperature in the trachea. Results are presented as the mean ± SD (n = 5).

Figure S3. Changes in cough reactivity to inhaled cinnamaldehyde and capsaicin in guinea pigs with cough induced by chronic inhalation of citric acid following exposure to a cold environment (0°C) with or without the pretreatment with 1.6 mM of HC030031. Results are presented as the mean ± SD (n = 5; * p < 0.05, ** p < 0.01, *** p < 0.001).

Figure S4. Effect of a vehicle solution and different concentrations of HC-030031 on cough reactivity to inhaled cinnamaldehyde in guinea pigs with cough induced by chronic inhalation of citric acid. Results are presented as the mean ± SD (n = 5; * p < 0.05).

Figure S5. Different expression levels of TRPA1 protein in the skin of various parts of guinea pigs. Results are presented as the mean ± SD (n = 5; * p < 0.05).
